# Guidelines for planning genomic assessment and monitoring of locally adaptive variation to inform species conservation

**DOI:** 10.1111/eva.12569

**Published:** 2017-12-02

**Authors:** Sarah P. Flanagan, Brenna R. Forester, Emily K. Latch, Sally N. Aitken, Sean Hoban

**Affiliations:** ^1^ National Institute for Mathematical and Biological Synthesis University of Tennessee Knoxville TN USA; ^2^ Duke University, Nicholas School of the Environment Durham NC USA; ^3^ Department of Biological Sciences University of Wisconsin‐Milwaukee Milwaukee WI USA; ^4^ Faculty of Forestry University of British Columbia Vancouver BC Canada; ^5^ The Morton Arboretum Lisle IL USA; ^6^ Present address: Department of Biology Colorado State University Fort Collins CO USA

**Keywords:** adaptive management, conservation genetics, conservation planning, local adaptation, natural selection, next‐generation sequencing, outlier detection

## Abstract

Identifying and monitoring locally adaptive genetic variation can have direct utility for conserving species at risk, especially when management may include actions such as translocations for restoration, genetic rescue, or assisted gene flow. However, genomic studies of local adaptation require careful planning to be successful, and in some cases may not be a worthwhile use of resources. Here, we offer an adaptive management framework to help conservation biologists and managers decide when genomics is likely to be effective in detecting local adaptation, and how to plan assessment and monitoring of adaptive variation to address conservation objectives. Studies of adaptive variation using genomic tools will inform conservation actions in many cases, including applications such as assisted gene flow and identifying conservation units. In others, assessing genetic diversity, inbreeding, and demographics using selectively neutral genetic markers may be most useful. And in some cases, local adaptation may be assessed more efficiently using alternative approaches such as common garden experiments. Here, we identify key considerations of genomics studies of locally adaptive variation, provide a road map for successful collaborations with genomics experts including key issues for study design and data analysis, and offer guidelines for interpreting and using results from genomic assessments to inform monitoring programs and conservation actions.

## 1 INTRODUCTION

Natural selection is a powerful force that can shift the genetic makeup of a population through time, increasing average fitness of individuals. Some adaptations, such as resistance to a widespread disease, will increase fitness of individuals in most or all populations of a species, while other adaptations are advantageous only under certain local environmental conditions, termed **local adaptation** (Box 1). Information on the extent and nature of local adaptation can be used by managers to inform conservation actions to improve the evolutionary potential and adaptive capacity of populations under the diverse stressors imposed by changing environments (Box 2). For example, the success rate of restoration and reintroduction efforts can be improved by matching genotypes to current or future environmental conditions. In reforestation efforts, trees of local provenance outperform those from distant seed sources, with greater survival, health, and productivity due to local adaptation to climate (Aitken & Bemmels, 2016; Langlet, 1971). By contrast, if local adaptation exists but is not accounted for, restoration and reintroduction may be less successful because individuals fail to thrive under the local environmental conditions. This outcome wastes resources and may cause negative ecological impacts. For example, sowing poorly adapted seed from native plant species in the Great Basin has resulted in poor establishment despite a high price tag (Kulpa & Leger, 2013; Leger & Baughman, 2014; Rowe & Leger, 2012). Genetically based heat tolerance may be similarly crucial for restoring or managing fisheries and coral systems (Jensen et al., 2008; van Oppen, Oliver, Putnam, & Gates, 2015). In situations like these, identifying geographic patterns of local adaptation informs and improves conservation actions.

Box 1 **Definitions**

1 **Adaptive management:** A structured decision‐making framework for problems where decisions are recurrent and uncertainty is an impediment to action (Runge, 2011).

**Bioinformatics:** A scientific field at the intersection of mathematics, computer science, and statistics, which develops methods and software for analyzing and interpreting complex biological data. Bioinformatics is commonly used to analyze large next‐generation sequencing datasets.

**Common garden:** An experimental approach in which organisms from two or more different environments are moved from their native environment into a common environment and reared through an entire life cycle under the same conditions. Traits are compared among individuals from different native environments to determine whether there is a genetic component to the differences among environments.

**De novo assembly:** Sequence reads are assembled without the aid of a reference genome. Instead, sequence reads are assembled into contigs (overlapping sequences that are nearly identical) and scaffolds (sets of contigs oriented approximately in relation to each other). Quality of de novo assemblies is assessed using metrics including the length of the contigs and the degree of sequence overlap. De novo assembly is common in studies of nonmodel organisms where reference genomes from the focal species or related species are not available.

**Effective population size (*N*_*e*_):** The size of an ideal, randomly mating population that experiences genetic drift at the same rate as the census population (*N*_*c*_). Typically, *N*_*e*_ is smaller than *N*_*c*_ due to processes that accelerate drift such as nonrandom mating, unequal reproductive success, and fluctuating population sizes. *N*_*e*_/*N*_*c*_ is often 1/10 to 1/4, but sometimes much smaller. To simplify slightly, *N*_*e*_ is approximately the number of individuals in a population who contribute to offspring in the next generation.

**Exome:** The subset of the genome that is composed of exons, the parts of genes that are transcribed after RNA splicing occurs (i.e., sequence data not including introns or other noncoding regions of the genome).

**Genetic drift:** A change in allele frequencies over time due to stochastic processes (random transmission from generation to generation). Drift occurs in all populations but operates more quickly in small populations (*N*_*e*_ ≤ 1,000, although there is debate on the exact threshold). Drift decreases genetic variation and drives alleles toward fixation (frequency of 0 or 1).

**Genetic markers:** Any type of genetic sequence information that can be used to identify differences between individuals, populations, and/or species. Examples include (but are not limited to) microsatellites, fragment length polymorphisms, single nucleotide polymorphisms, and gene sequences.

**Genomic:** A loosely defined term that can refer to the use of large numbers of anonymous genetic markers (thousands to millions), the use of targeted gene sequences, or analyses that account for genomic context such as linkage, recombination, or gene function (Allendorf et al., 2010; Garner et al., 2016). The distinction between “genetic” and “genomic” studies varies across the literature. Here, we differentiate genetic studies as those using smaller sets of markers that can be treated as independent, whereas genomic studies use many markers that are no longer presumed to be independent loci. Most genetic studies address questions related to neutral processes (e.g., gene flow, genetic drift), while genomic studies often address questions related to local adaptation, selection, and ecologically relevant traits. Due to the large number of markers produced by genomic studies, questions related to neutral processes are also frequently addressed, providing greater resolution than genetic studies.

**Indicator variable:** A variable that is being monitored, such as heterozygosity. When the indicator variable reaches a trigger point, a predefined conservation action will be taken which aims to bring the indicator variable back below the threshold.

**Linkage:** A statistical association between two genetic markers that arises due to the markers being physically located near each other on a chromosome, close enough that recombination between the two markers is unlikely. Genetic markers may exhibit statistical linkage if they are inherited together (i.e., do not independently assort), even if they are not physically proximal.

**Local adaptation:** Due to the action of natural selection, resident genotypes have higher relative fitness in their local environment than genotypes from other environments.

**Microsatellites:** Anonymous markers whose alleles are defined by polymorphism in the length of the DNA sequence. Microsatellite markers have many different alleles (in comparison with biallelic SNPs), meaning that genetic variation can be captured by fewer microsatellite markers than would be captured by the equivalent number of SNPs. Therefore, most microsatellite studies have fewer than 30 markers, compared to more than 1,000 markers for studies using SNPs. However, this low number of markers does not provide sufficient genomewide coverage for estimating genomewide parameters.

**Reciprocal transplant:** An experimental approach in which organisms from two different environments are raised in both environments. Traits are compared between environments to determine whether there is a genetic component to the differences between environments (adaptive differentiation).

**Recombination:** The exchange of genetic material either between multiple chromosomes or between different regions of the same chromosome. Recombination typically occurs during meiosis, when homologous chromosomes pair up to be passed on to the gametes (this process is also referred to as “crossover”).

**Sensitivity analysis:** The process of testing a variety of parameter settings using the same starting data (e.g., raw reads) to compare the results from different parameter combinations. If the results from different parameter settings are qualitatively similar, then the results are likely a real signal. If the data are highly sensitive to parameter settings, it might be worth investigating to see whether there is a major source of bias in the dataset.

**Single nucleotide polymorphism (SNP):** One base pair in a DNA sequence that shows variation among individuals. SNPs are typically biallelic (have only two alleles) and occur frequently throughout genomes.

**Transcriptome:** The set of messenger RNA transcripts that are produced in a cell or tissue in response to factors such as the environment or developmental stage. To generate sequencing data for these messenger RNA transcripts, RNA from a particular tissue is converted to DNA and sequenced in short reads on high‐throughput sequencing machines (e.g., Illumina machines). These short reads are then bioinformatically assembled to create sequences for genes; these consensus gene sequences are the “transcriptome.”

**Trigger point:** A value for an indicator variable that is decided before monitoring begins. When the indicator variable reaches this point, a predefined conservation action will be implemented.

Box 2 **Conservation actions benefiting from knowledge of local adaptation**

1 Identifying geographic patterns of local adaptation, the environmental drivers of divergent selection among populations, and genes and their variants involved in local adaptation can inform conservation strategies for species at risk (Allendorf et al., 2010; Shafer et al., 2015), especially in the context of changing environmental conditions (global changes in climate or local changes in land use, fire, hydrology, and other processes altering a species’ local habitat). Genetic variants that help individuals within populations survive or reproduce more under new environmental conditions would be considered adaptive. If adaptive genetic variants are identified, individuals with genotypes more likely to have higher fitness in local environments could be used in breeding, reinforcement, or reintroduction programs to help ensure success of those programs (He, Johansson, & Heath, 2016; Kelly & Phillips, 2016; Sgro et al., 2011). Managers could also monitor the frequency of these genetic variants over time to gauge the genetic health of a population, or to assess changes in allele frequencies following management interventions (Schwartz et al., 2007; Shafer et al., 2015).

Although adaptive genetic variation is an important consideration for conservation actions, it is clear that managing for specific adaptive variants without regard to genetic variation across the rest of the genome should generally be avoided (Pearse, 2016), unless such variants are well verified by other evidence (e.g., aridity tolerance in eucalyptus; Steane et al., 2014) and the situation is urgent (e.g., disease progression). Even in cases where the evidence for genetic adaptation is strong, management interventions should strive to conserve adaptive variation without eroding genomewide variation (Giglio, Ivy, Jones, & Latch, 2016; Haig, Ballou, & Derrickson, 1990; Spielman, Brook, & Frankham, 2004). Conversely, management actions designed to preserve genomewide variation may either involve risks of disrupting local adaptation to nonclimatic factors (e.g., biotic interactions, soils) if local adaptation is not well understood, or could result in outbreeding depression if individuals from long‐diverged populations are mixed and interbreed (see Frankham et al., 2011 for guidance on when this might occur). However, many conservationists argue that the benefits of introducing needed genetic variation for challenging environmental conditions may outweigh these risks (Aitken & Whitlock, 2013; Whiteley, Fitzpatrick, Funk, & Tallmon, 2015).

Below we provide some specific conservation actions that would benefit from the inclusion of assessment and monitoring of adaptive variation.

**Assisted gene flow:** Assisted gene flow is the movement of individuals within the species range from an adaptively divergent source population that has genetic variation predicted to be adaptive under future environmental conditions (Aitken & Whitlock, 2013; Prober et al., 2015). NGS can be used to characterize local adaptation based on environmental conditions. Then, “preadapted” genetic variants from a source population can be moved into a recipient population to improve evolutionary potential. While appropriate source and recipient populations could be selected based on climatic and other ecological data (a “best guess” approach), such efforts would be better informed by knowledge of adaptive variation and climatic drivers of local adaptation. Assisted gene flow is expected to be especially beneficial in dispersal‐limited, long‐lived species such as trees (Aitken & Bemmels, 2016; Gugger, Liang, Sork, Hodgskiss, & Wright, 2017; Steane et al., 2014).

**Defining conservation units:** Starting in the 1990s, a few (5–25) selectively neutral markers (e.g., microsatellites and organellar DNA) were commonly used to delineate conservation units. NGS provides increased resolution, while also allowing for characterization of adaptive differentiation among populations. Funk, McKay, Hohenlohe, and Allendorf (2012) explain how to use both neutral and adaptive data in a complementary way to delineate conservation units that maximize adaptive capacity, while Ahrens et al. (2017), Guo, Li, and Merilä (2016), Lah et al. (2016), and Peters et al. (2016) provide empirical examples.

**Environmental epidemiology and disease dynamics:** NGS can be used to investigate the genetic basis of disease, parasite, and toxin resistance. This is a relatively underutilized application of NGS in wild populations, although a few excellent examples exist, including identifying the genetic basis of adaptation to harmful algal blooms in coastal and estuarine common bottlenose dolphins (Cammen, Schultz, Rosel, Wells, & Read, 2015), and identifying a rapid evolutionary response to transmissible cancer in multiple populations of Tasmanian devils (Epstein et al., 2016).

**Genetic rescue:** The aim of genetic rescue is to improve the fitness of small populations by increasing (neutral) genetic diversity by moving individuals between populations (Whiteley et al., 2015). The main concern with genetic rescue is outbreeding depression, a reduction in fitness due to the mixing of divergently adapted genotypes and/or the disruption of co‐adapted gene complexes. Adaptive markers identified with NGS can characterize adaptive differences among source and target populations, while neutral markers can be used to estimate the extent of gene flow between these populations. This information can then be used to minimize the risk of outbreeding depression. See Weeks et al. (2011) for a definitive discussion.

**Identifying hybridization:** Although not strictly a conservation action, identifying hybrids has direct relevance for conservation managers, because hybridization can be both a conservation problem, threatening species identity and genetic integrity (Bohling, 2016; Wayne & Shaffer, 2016), and a conservation opportunity, enhancing evolutionary potential in changing environments through adaptive introgression (Hamilton & Miller, 2016). In both cases, NGS provides both improved resolution to identify hybridization and the data needed to develop monitoring panels (Hohenlohe et al., 2011).

**Minimizing adaptation to captivity:** Although no examples are published to date, adaptive NGS could be used in captive breeding programs to monitor for rapid changes in allele frequencies that could be indicative of adaptation to captive conditions (Allendorf et al., 2010), which can have severe fitness consequences for reintroduced populations (Black, Seears, Hollenbeck, & Samollow, 2017).

**Site prioritization to maximize evolutionary potential:** Site prioritization conventionally involves maximizing the amount of biodiversity protected (e.g., number of species) while minimizing financial costs. Under climate change, protecting populations with complementary sets of intraspecific adaptive genetic diversity has become increasingly important, as this adaptive variation is indicative of the evolutionary potential of populations under changing conditions (Bonin, Nicole, Pompanon, Miaud, & Taberlet, 2007). NGS can provide both the neutral and adaptive data needed for these analyses.

While the traditional method for testing local adaptation is to assess the relative survival and fitness of populations in **reciprocal transplant** or **common garden experiments**, this is costly, time‐consuming, and often not feasible for species at risk. Another complementary approach that can be used in any species is to screen large numbers of **genetic markers** to identify variation associated with environmental factors or adaptive traits. These analyses, made possible due to advances in genetic sequencing technologies (i.e., next‐generation sequencing, NGS), provide unprecedented opportunities to integrate **genomic** data into conservation management of nonmodel species (Harrisson, Pavlova, Telonis‐Scott, & Sunnucks, 2014; Hoffmann et al., 2015). However, genomic studies of local adaptation are not appropriate, informative, or necessary in all cases (Allendorf, Hohenlohe, & Luikart, 2010). Additionally, despite falling costs, these studies still require significant financial and computational resources, as well **bioinformatics** expertise.

**Figure 1:**
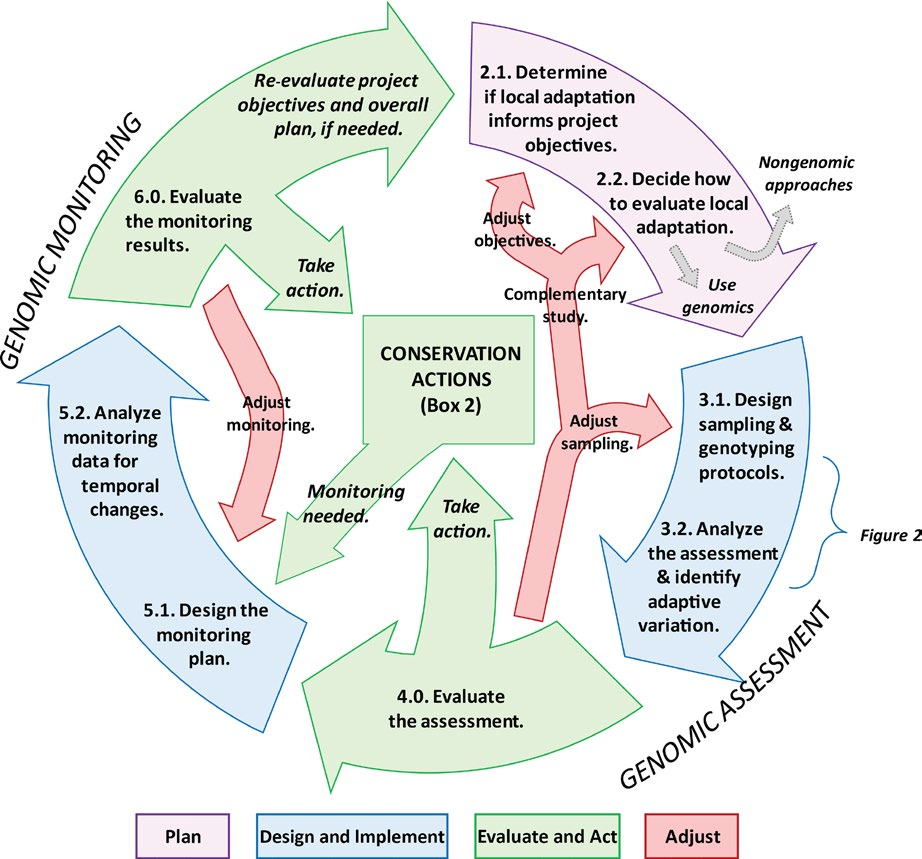
Adaptive management cycle for NGS‐based assessment and monitoring of adaptive genetic variation. Cycle stages numbered to match sections in the text. Stage 2 outlines the initial planning phase, stages 3 and 4 are the genomic assessment, and stages 5 and 6 are the genomic monitoring phases. The red, un‐numbered arrows highlight the need for adjusting the plan throughout the adaptive management cycle

Several reviews already exist on the *potential* of using genomic data to detect adaptive variation for conservation purposes (Allendorf et al., 2010; Harrisson et al., 2014; Hoffmann & Sgro, 2011; Hoffmann et al., 2015; Sgro, Lowe, & Hoffmann, 2011; Stapley et al., 2010; Stillman & Armstrong, 2015). Here, we aim to provide a *guide* to help conservation biologists and managers decide whether using genomics to detect local adaptation is an appropriate investment, as well as a road map for successful collaboration with genomics experts. We emphasize the iterative and challenging nature of studies of adaptive variation and the specific need for monitoring programs that are linked to conservation actions, which are often characterized by high uncertainty. We also describe situations when identifying local adaptation using genomic approaches is not likely to be useful. We use a modified **adaptive management** framework (Runge, 2011; Williams & Brown, 2016) to highlight the important steps in a genomic study of adaptive variation that includes both assessment and monitoring (Figure 1): Plan, Design and Implement, Evaluate and Act, and Adjust. A key distinction we make within this framework is between genomics‐based *assessment*, which is a point‐in‐time evaluation to identify existing adaptive variation in the populations or species of interest, and population genetic or genomics‐based *monitoring*, which has a temporal component to monitor change (Schwartz, Luikart, & Waples, 2007). In most cases, as reflected in Figure 1, monitoring protocols will be developed from the initial genomic assessment. The best results will come from team members (ecologists, geneticists, bioinformaticians, conservation managers) working together through the entire adaptive management cycle and sharing their expertise while communicating uncertainties, practicalities, and assumptions to other team members.

## 2 PLAN: INCLUDING ADAPTIVE VARIATION

### 2.1 Determine whether knowledge of local adaptation informs conservation objectives

Many projects with conservation goals can be informed by knowledge of local adaptation (Box 2). In some cases, such as assisted gene flow (Box 2), incorporating adaptive variation into the assessment plan is a primary objective (Aitken & Whitlock, 2013). Alternatively, conservation goals may be adequately addressed using neutral genetic variation (e.g., to infer demographic parameters), and data on adaptive variation may be unnecessary or secondary to the project. For example, assessment and management of inbreeding through genetic rescue only requires knowledge of neutral variation, although an understanding of local adaptation may reduce the risks of outbreeding depression by minimizing adaptive divergence between source and target populations (Box 2).

Several features of species and their populations should be considered when determining whether to use genomic approaches to study adaptive variation. Species where local adaptation is most likely to occur and be detected using genomics are characterized by strong environmental variation among populations (producing divergent selection), and large **effective population size** (minimizing the effects of **genetic drift**). When divergent selection is strong, local adaptation is likely to develop, even in the face of high gene flow (Yeaman & Whitlock, 2013). Signatures of local adaptation are more likely to be detected in species with minimal neutral population structure, such as mobile species with high gene flow (common in marine systems), because strong population structure or complex evolutionary history can create many false positives (De Mita et al., 2013; Lotterhos & Whitlock, 2015; de Villemereuil, Frichot, Bazin, François, & Gaggiotti, 2014). By contrast, local adaptation is less likely in systems with homogenous environmental conditions or where environmental conditions fluctuate over time. Local adaptation is also less likely in populations with small or highly variable effective sizes (where genetic drift has stronger effects). Very low levels of gene flow can lead to strong neutral population structure that can make it difficult to distinguish patterns due to selection from those resulting from demography. If managers are working with species that have characteristics making local adaptation less likely to develop or to be detectable, and where there is no prior evidence of local adaptation, managers might consider allocating scarce resources to other conservation activities, rather than investing in genomic methods that may produce ambiguous results.

### 2.2 Decide how to evaluate local adaptation

If the project will benefit from understanding local adaptation, several options exist. For species that are amenable to experimental approaches (e.g., plants), patterns of local adaptation can be reliably addressed by traditional methods such as common gardens and reciprocal transplants (Blanquart, Kaltz, Nuismer, & Gandon, 2013; Endler, 1986; Hereford, 2009; Kawecki & Ebert, 2004). Longer‐term field studies of wild populations can also be used to assess adaptive variation in some contexts (Charmantier, Doutrelant, Dubuc‐Messier, Fargevielle, & Szulkin, 2016; Charmantier et al., 2008; Ozgul et al., 2009). For example, in Mediterranean blue tits, egg laying date is heritable and differs between populations in deciduous and evergreen forests, and those differences are maintained in common garden conditions (Charmantier et al., 2016). These types of studies may be more affordable and can be just as effective as genomic approaches in providing necessary information on local adaptation. While transplantation or long‐term studies are not possible for all species of conservation concern, it will be an option for some, including many plants (McKay et al., 2001; Raabová, Münzbergová, & Fischer, 2007).

In many cases, however, phenotypic methods will not be feasible for the focal species, and genomics may be the preferred alternative. Many management issues related to local adaptation do not require a complete assessment of adaptive variation, nor the functional validation of candidate adaptive variants. Instead, managers may simply need to characterize geographic or environmental patterns of adaptive variation across populations, information which can be generated for species without prior genomic information (Catchen et al., 2017). However, there are advantages to working with species that already have some genomic resources developed (sometimes called a “genome‐enabled” species; Kohn, Murphy, Ostrander, & Wayne, 2006), such as an assembled reference sequence or **transcriptome**. These resources maximize useable data and can help validate and interpret potentially adaptive variation (e.g., by comparing to genes with known function). Additionally, any genomic study is more difficult (e.g., laboratory protocols will require more troubleshooting and modification) and potentially costlier in species with large genomes (e.g., conifers, salamanders). Overall, before deciding to embark on a genomic study of adaptive variation, we recommend clearly defining the biological or management questions, identifying how genomic data will help address these questions, evaluating alternative nongenomic approaches, researching any existing genetic resources for the focal or a closely related species (or identifying whether those resources need to be developed), considering biological and genomic characteristics of study species, and evaluating budgetary constraints for both assessment and management.

## 3 DESIGN AND IMPLEMENT: ASSESSMENT

### 3.1 Design the sampling and genotyping protocols

**Figure 2:**
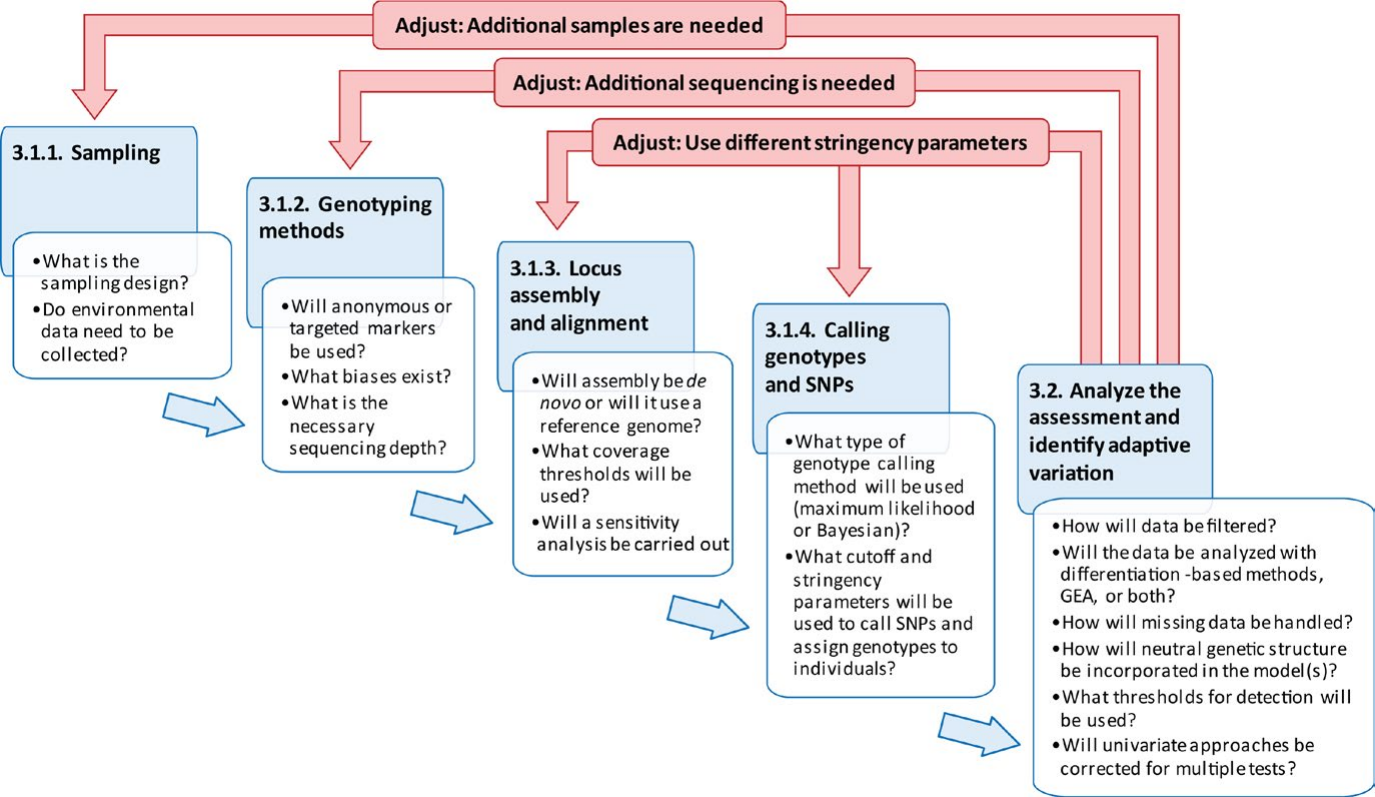
Key questions to ask when conducting a genomic assessment of adaptive variation. The steps here correspond to stage 3 in Figure 1. As in Figure 1, the red, un‐numbered arrows identify potential points where adjusting the planned assessment is required

In every genomics study, researchers make many small decisions about sampling, genotyping, bioinformatics, and analysis, all of which can have a substantial impact on downstream results. Managers should not be expected to know every detail, but some decisions, which we highlight in this section and in Figure 2, should be discussed carefully among the team members as they can impact the interpretation of the study.

#### 3.1.1 Sampling

Sample size and the number and location of populations sampled are primary considerations that can dramatically facilitate or impede detection of local adaptation. All methods for detecting local adaptation will benefit from sampling that is stratified across environmental gradients likely driving selection and replicated across those gradients (Lotterhos & Whitlock, 2015; Schoville et al., 2012). How individual samples are specifically arrayed (e.g., individual‐ or population‐based sampling, number of individuals per population, transects, or paired designs) is less generalizable and depends on the analytical approaches to be used and the biology and distribution of the species. For example, many genotype–environment association (GEA) methods for detecting local adaptation can be used on either individual or pooled population samples, while differentiation‐based approaches require population‐based sampling (see below). Researchers will often try to accomplish multiple goals when collecting genomic data (e.g., estimate effective population size, inbreeding, gene flow, and adaptive differentiation), and characterizing adaptive variation may be only one of several objectives. One sampling plan may not fit all objectives; it is therefore important to plan ahead and target sampling to meet primary objectives, while consulting with collaborators on how data may be used to meet secondary goals. For this reason, sampling will involve trade‐offs, including accommodating multiple analytical goals, achieving sufficient geographic coverage to sample known or suspected genetically differentiated populations, sufficiently sampling the environmental conditions thought to be driving selection, sufficiently replicating sampling along environmental gradients, and sampling sufficient numbers of locations and individuals per location. For example, De Mita et al. (2013) showed via simulation that relatively good performance could be achieved with at least eight sampled populations, using a strategy that samples the extreme ends of the environmental gradient, but the best sampling in real situations is not fully known.

Most genomics protocols (Etter, Bassham, Hohenlohe, Johnson, & Cresko, 2011; Peterson, Weber, Kay, Fisher, & Hoekstra, 2012; see below) require 50–300 ng of high‐quality DNA, taken from small (often nonlethal) tissue samples. Recent studies have successfully used NGS on as little as 1 ng of DNA extracted from noninvasively collected samples (i.e., hair snags) and museum samples, indicating that even low‐quality samples can be used, but do require additional precautions and genomic resources because DNA degrades over time (Bi et al., 2013; Russello, Waterhouse, Etter, & Johnson, 2015). These advances have the potential to extend local adaptation studies to species that are difficult to sample, and allow for the retrospective study of genetic variation.

For analyses that incorporate environmental variation, such as GEA, environmental sampling will also be required. Key environmental factors will depend on the focal species, and experts with in‐depth knowledge of species biology can suggest potentially important habitat (e.g., soil type, plant community, water quality, pollution) or climatic factors (e.g., seasonal and annual temperature and precipitation averages and extremes). Environmental characterization may be as simple as collecting weather station data or relevant GIS layers from online databases (see Daly 2006 for guidance on assessing the suitability of spatial climate datasets). In these cases, the temporal scale of environmental data should be considered in relation to the generation time of the species, such that environmental covariates include multiple generations of selective pressures. Researchers should also consider selection pressures that occur at specific life history stages, such as seedling establishment in long‐lived trees, which may experience different selective pressures than those observed in fully grown trees. When covariates such as environmental contaminants need to be measured directly in the field, additional planning is required (e.g., for instrument acquisition, deployment, maintenance, and data analysis). When available, it is best to use proximal (e.g., temperature, precipitation) as opposed to distal (e.g., elevation, latitude) predictors, as proximal variables may decouple from their distal proxies, for example, under climate change (Lookingbill & Urban 2005). Finally, consideration of environmental variability should be included with mean predictors, especially as temporal and spatial variability in climate may be magnified by climate change (Buckley & Huey, 2016; Reusch, Ehlers, Hammerli, & Worm, 2005; Schoepf, Stat, Falter, & McCulloch, 2015). Detailed genetic and environmental sampling guidelines are reviewed elsewhere (Balkenhol & Fortin, 2016; De Mita et al., 2013; Hoban et al., 2016; Lotterhos & Whitlock, 2015; Manel et al., 2010; Prunier et al., 2013; Rellstab, Gugerli, Eckert, Hancock, & Holderegger, 2015; Schoville et al., 2012).

#### 3.1.2 Genotyping methods

Genomic data are most often produced using NGS technologies that can sequence millions of DNA fragments across the genome (Davey et al., 2011; Goodwin, McPherson, & McCombie, 2016). In most cases, only a subset of the genome is sequenced. Two primary methods are used to reduce the amount of the genome sequenced: anonymous sequencing methods that sequence DNA adjacent to restriction enzyme cut sites, and targeted sequencing methods that focus on known genes or sequences. The most commonly used anonymous approaches in ecological and evolutionary studies are the family of restriction‐site‐associated DNA sequencing (RADseq) protocols, which include a diversity of library preparation methods (Andrews, Good, Miller, Luikart, & Hohenlohe, 2016). By contrast, targeted sequencing focuses on capturing specific genomic regions, ranging from specific neutral markers, to candidate genes to entire **exomes** (Grover, Salmon, & Wendel, 2012). Of the targeted sequencing methods, sequence capture is the most scalable to whole‐genome applications (Grover et al., 2012; Jones & Good, 2016) and is particularly useful for species with large genomes (Suren et al., 2016).

Anonymous and targeted sequencing methods have trade‐offs in cost, accuracy, and bias. Anonymous sequencing methods require no prior genomic information and less starting DNA and are usually considerably less expensive than targeted sequencing. However, depending on the protocol used, they are subject to problems with error, bias, and missing data. These issues include genotyping biases (e.g., false homozygosity) due to sources of error such as PCR bias (Davey et al., 2011), PCR duplicates (Davey et al., 2011), polymorphic restriction sites (i.e., allele dropout; Arnold, Corbett‐Detig, Hartl, & Bomblies, 2013; Cariou, Duret, & Charlat, 2016; Gautier et al., 2013), and shearing bias (Davey et al., 2013). Many of these issues are specific to particular RADseq protocols and can be addressed with appropriate planning and study design (for a review of problems, solutions, and RADseq study design, see Andrews et al., 2016; Catchen et al., 2017; Lowry et al., 2017a,b; McKinney, Larson, Seeb, & Seeb, 2017). Because RADseq genotypes a subsample of regions across the genome, it will include both selectively neutral and adaptive markers.

Targeted sequencing requires prior sequence resources (e.g., a transcriptome assembled from RNA sequencing, reference genome, or anonymous sequences) for the design of capture probes (Grover et al., 2012; Jones & Good, 2016). The success rate of sequence capture probes increases with the use of a reference genome for identifying intron–exon boundaries. If targets are designed based on a reference genome from another species, the suite of loci may be biased when applied to the focal species (a form of ascertainment bias), although aligning to a congener should reduce bias.

Regardless of the genome complexity reduction method used prior to sequencing, in most cases multiple individuals will be individually barcoded, then pooled in a lane of sequencing. Because of error and bias that can arise from library preparation and sequencing, randomizing samples throughout the process is instrumental in reducing bias (Meirmans 2015). Individuals from the same populations or from nearby locations should be distributed among sample plates and sequencing libraries. Otherwise, estimates of population genetic statistics may be biased.

Decisions on whether to use anonymous or targeted sequencing should be based on the overall study goals and the availability of prior genomic resources. As total gene content does not vary as much as genome size, anonymous sequencing will be relatively poorer for detecting adaptive variation in species with larger genomes, as fewer sequences will contain coding regions, and more missing data will result from sequencing efforts scattered over a larger number of sequences (Lowry et al., 2017a,b). Prior to choosing a sequencing method, researchers and managers should discuss and be aware of biases and sources of error that will impact the downstream analyses.

#### 3.1.3 Assembly and alignment of sequence reads

Next‐generation sequencing generates many short sequence reads that need to be assembled into groups of similar, homologous sequences and then aligned to a genomic location within a reference genome (if one is available). Polymorphic loci are then identified and the genotypes of individuals inferred from their reads for these loci (described in Section 3.1.4). In targeted sequence capture, probes are often designed for exons of known genes. In anonymous sequencing methods, sequenced regions are scattered across the genome in introns and exons within genes, but also in intergenic regions, and so are more vaguely referred to as “loci.” Here, we will use the term “loci” to refer to sequenced regions used in the analyses for simplicity.

For anonymous sequencing approaches, an important decision is whether to use a reference genome to guide the assembly of loci or to conduct a **de novo assembly** with the sequence data. This choice will determine the appropriate type of assembly program to use (e.g., GATK: McKenna et al., 2010; dePristo et al., 2011; Van der Auwera et al., 2013 with a reference genome; Stacks: Catchen, Amores, Hohenlohe, Cresko, & Postlethwait, 2011; Catchen, Hohenlohe, Bassham, Amores, & Cresko, 2013; Paris, Stevens, & Catchen, 2017; or dDocent: Puritz, Hollenbeck, & Gold, 2014 for a de novo assembly). Using a high‐quality and well‐annotated reference genome facilitates the identification of candidate genes and gene regions and allows for a truly genomic approach (e.g., considering physical linkage between regions with adaptive variation; Manel et al., 2016). However, using a reference genome from another species can also result in confirmation bias, because the focal species may have divergent gene sequences or different structural features of the genome that may result in informative loci being removed from the analysis (Tamazian et al., 2016). Developing a high‐quality reference genome for the focal species would ameliorate some of these issues, but is not always necessary, depending on objectives. Managers should be aware of whether a reference genome is available, and whether it is for the focal species or a congener.

A major decision that will determine which loci are included in the dataset is choosing the parameters determining how closely the sequences must match (either match the reference sequence or match other sequences in de novo approaches; Catchen et al., 2011; McKenna et al., 2010; dePristo et al., 2011; Van der Auwera et al., 2013) and how often the sequences occur in individuals (i.e., coverage). If the sensitivity of these parameters is too low, sequences will be combined that are not from the same genomic region (i.e., paralogs; McKinney, Waples, Seeb, & Seeb, 2017). Alternatively, if settings are too stringent, few loci will be included. To help identify the best parameters and understand the limitations of the dataset, **sensitivity analysis** should be performed (Andrews & Luikart, 2014; Escudero, Eaton, Hahn, & Hipp, 2014; Mastretta‐Yanes et al., 2015; Paris et al., 2017). Biases identified by sensitivity analysis, such as a large number of PCR duplicates or excessive missing data, may be addressed through more stringent filtering, or it may be necessary to collect more data (resequencing, sampling more individuals, or considering another sequencing approach). For anonymous methods, including technical replicates (i.e., using the same DNA but barcoding and processing the replicate independently) in the genotyping library is recommended to improve quality control (e.g., estimating error rates) and parameter optimization (Mastretta‐Yanes et al., 2015).

#### 3.1.4 Calling genotypes and SNPs

Once loci are selected for analysis, sequence reads spanning each locus from each individual are used to call genotypes (i.e., infer the genotype at a locus for each individual; Nielsen, Paul, Albrechtsen, & Song, 2011). Genotype‐calling software programs use either maximum‐likelihood (e.g., Stacks; Catchen et al., 2011) or Bayesian models (e.g., GATK; McKenna et al., 2010; dePristo et al., 2011; Van der Auwera et al., 2013) to assign individuals with genotypes. These models often incorporate some element of sequencing error, but the primary determinant of whether individuals are accurately genotyped as heterozygous or homozygous is the number of reads assigned to each individual. While most polymorphisms will be SNPs, one major consideration when grouping reads into exon regions (applicable when a reference is available) is identifying and correctly aligning insertion and deletion mutations (INDELs). The importance of correcting for INDELs in accurate SNP calling depends on the mapping and calling programs used (O'Rawe et al., 2013).

Similar to filtering polymorphic loci for analysis in the dataset, the thresholds set for SNP calling for individuals influence the quality of the data (Nielsen et al., 2011). For example, if the dataset contains too few sequences for an individual across a given SNP, an individual that is a heterozygote may be wrongly genotyped as a homozygote if only one of the two alleles is sequenced. Software programs typically allow the user to specify coverage cutoffs and other parameters determining SNP calling stringency. Changing the parameters of these models, especially the number of reads required to call heterozygotes, can affect genotypic frequencies in the populations and alter population genetics statistics estimated in the analyses. Depending on the depth of coverage, this threshold can also reduce the size of the dataset (Huang & Knowles, 2014). In exome capture studies, quality control that is too stringent can lead to a loss of power if causal variants are removed (Auer, Wang, & Leal, 2013). An additional consideration is whether the phased haplotype within a locus can be analyzed instead of single SNPs (Benestan et al., 2016; Manching et al., 2017). Many loci have multiple SNPs within an exon or locus, and those SNPs can be combined to infer a haplotype (Helyar et al., 2011). Additionally, if a reference genome is available, the position of the SNPs in a broader genomic region can be used to infer haplotypes (Andolfatto et al., 2011; Andrews et al., 2016). However, many of the common and user‐friendly downstream analytical programs only consider independent SNPs.

To summarize, we encourage conservation managers to become familiar with the primary steps that can influence data quality and interpretation of results. When planning a project, based on the objectives of the project, the team must decide (i) which NGS method will be used; (ii) whether a reference genome is available; (iii) how the genotype‐calling coverage and mismatch thresholds will be set, and whether the sensitivity of the data to those parameters will be evaluated; and (iv) what coverage cutoffs will be used to select loci and assign genotypes to individuals (Figure 2).

### 3.2 Analyze the genomic assessment and identify adaptive variation

The first step in analyzing genotypic data collected during the assessment is quality control filtering. Data filtering is a multistep process, with specific criteria dependent on the analyses to be performed (see Benestan et al., 2016 for a recent overview). Quality control filters are used to ensure that uninformative markers and statistical artifacts are removed prior to analyses. These filters consider sequencing error, locus coverage, genotyping level (across loci, individuals, and populations), number of alleles per marker, and linkage (e.g., number of SNPs per genomic contig or exon). Filters may also be applied based on minor allele frequency and deviations from Hardy–Weinberg proportions. These filters can reduce the size of the dataset, but increase the quality of the analysis (Huang & Knowles, 2014). Patterns of missing data across samples should also be evaluated both before and after filters are applied to reduce the risk of detecting spurious (nonbiological) signals in downstream analyses. This includes visualizing relationships between missingness and factors such as sequencing lane, sample site, population, and heterozygosity (Gosselin & Bernatchez, 2016). These visualizations can help determine if populations or individuals should be excluded, for example, if they have both high missing data and elevated homozygosity, suggesting allele dropout (i.e., one allele is not being sequenced). In some cases, populations may need to be resampled or samples resequenced to compensate for missing data (Figure 2).

Many methods for identifying local adaptation require a dataset without missing values, so missing data must either be pruned (e.g., removing loci or individuals) or imputed. The impact of these different strategies on downstream analyses is an area of active investigation (e.g., Chattopadhyay, Garg, & Ramakrishnan, 2014). Research in related fields indicates that strict filtering of missing data can reduce statistical power (Nakagawa & Freckleton, 2008), undermine inferential accuracy (Dai, Ruczinski, LeBlanc, & Kooperberg, 2006), and introduce bias (Huang & Knowles, 2014). With a lack of firm guidelines for anonymous sequencing data, which tends to have relatively high levels of missing data, the best current approach is to perform a sensitivity analysis using different filtering and imputation strategies. Gosselin and Bernatchez (2016) provide a large (and growing) set of imputation methods for anonymous sequencing data.

Methods for identifying candidate adaptive loci from genomic data can be divided into two main approaches, those based on population genetic differentiation (e.g., *F*_ST_ outlier methods) and genotype–environment associations (GEAs). These approaches, recently reviewed in Hoban et al. (2016) and Rellstab et al. (2015), differ in their data requirements and assumptions, and also in the information they generate for conservation planning. A third method associates genotypes with phenotypic traits involved in local adaptation to identify adaptive SNPs (i.e., genomewide association studies; reviewed in Savolainen, Lascoux, & Merilä, 2013), but we do not cover this method as sufficient phenotypic data are often unavailable for species of conservation concern. Differentiation‐based methods identify loci with extreme allele frequency differences among populations relative to overall population structure, a pattern consistent with divergent selection. These studies can be performed without prior knowledge of the environmental factors driving local adaptation and for species that exist in discrete populations, but often lack a specific hypothesis and will not identify environmental drivers of selection. Results are dependent on assumptions about the underlying distribution of selectively neutral differentiation (e.g., *F*_ST_) across loci. Some commonly used methods include tests based on the island model of migration as proposed by Beaumont and Nichols (1996) and implemented in LOSITAN (Antao, Lopes, Lopes, Beja‐Pereira, & Luikart, 2008), Mcheza/DFDIST (Antao & Beaumont, 2011), Arlequin (Excoffier & Lischer, 2010), and BayeScan (Foll & Gaggiotti, 2008). However, these methods are sensitive to deviations from the assumptions of the infinite island model (Flanagan & Jones, 2017; Hohenlohe, Phillips, & Cresko, 2010; Lotterhos & Whitlock, 2015) and are increasingly discouraged for empirical studies. Alternative approaches test other population genetic models (e.g., deviation from random genetic drift; Vitalis, Glemin, & Olivieri, 2004), relax the assumptions of a specific model (Lotterhos & Whitlock, 2015), or use methods that do not rely on population genetic models, such as principal components analysis (e.g., pcadapt; Luu, Bazin, & Blum, 2017).

By contrast, GEA methods identify potentially adaptive loci based on associations between allele frequencies and environmental variables hypothesized to drive selection, a pattern that is consistent with a selective advantage of certain alleles in certain environments (Joost et al., 2007). Unlike differentiation‐based approaches, these methods do not use an underlying population genetic model, and most can use either individual genotype or population allele frequency data. These methods generally have higher power than differentiation‐based methods, and can detect divergent selection even when it does not produce strong differentiation among populations (De Mita et al., 2013; Rellstab et al., 2015; de Villemereuil et al., 2014). Most GEA methods use some form of statistical control for population structure and demography, which, when unaccounted for, can produce high false‐positive signals (Hoban et al., 2016; Rellstab et al., 2015), although adjustments for population structure, especially when it is concordant with environmental gradients, can produce false negatives (e.g., Yeaman et al., 2016). Additionally, because most commonly used GEA methods (e.g., Bayenv2: Coop, Witonsky, DiRenzo, & Pritchard, 2010; Gunther & Coop, 2013; latent factor mixed models (LFMM): Frichot, Schoville, Bouchard, & François, 2013) use a univariate statistical framework in which one locus and one environmental predictor are tested at a time, these methods require corrections for multiple tests to prevent elevated false‐positive rates (François, Martins, Caye, & Schoville, 2016). Multivariate GEAs (e.g., redundancy analysis), which analyze many loci and environmental predictors simultaneously, identify how groups of loci covary in response to environmental predictors and may reduce or eliminate the need for multiple testing while potentially identifying polygenic selection (Rellstab et al., 2015). In simulations, multivariate GEAs are more effective than univariate methods at detecting important adaptive processes that result in weak multilocus signatures (e.g., selection on standing genetic variation) and are robust to multiple sampling designs and sample sizes (Forester, Lasky, Wagner, & Urban, 2017). Brauer, Hammer, and Beheregaray (2016) provide a clear example of local adaptation in a threatened fish species that is mediated by both divergent selection (detected through differentiation‐based methods) and polygenic selection from standing genetic variation (detected with a multivariate GEA).

For all of these methods of detecting locally adaptive variation, we recommend considering four key points: (i) Do the data meet the model assumptions? (ii) How is neutral genetic structure incorporated into the model? (iii) Are univariate approaches corrected for multiple testing? And (iv) what are the thresholds for detection? Thresholds for differentiating loci potentially under selection are generally arbitrary (e.g., FDR = 0.1) and should be tested and modified based on the study goals (François et al. 2016, de Villemereuil et al., 2014; Figure 2).

Conservation managers also must evaluate the risks of acting based on type 1 errors (concluding populations are not locally adapted when they actually are) from the risk of type 2 errors (concluding they are locally adapted when they are not), as different sequencing and analytical approaches carry different type 1 and type 2 risks. For example, if the proposed conservation action is genetic rescue, then acting on type 1 error increases the risk of outbreeding depression, whereas acting on type 2 error would minimize the number of available source populations. The conservation team can evaluate the risks of each type of error through sensitivity analysis. While to our knowledge, sensitivity analyses have not yet been used in applications of adaptive genomics in management, the benefit of these analyses is clearly evident in other aspects of conservation planning, including climate change vulnerability assessments (Wade et al., 2017), systematic conservation network planning (Levin, Mazor, Brokovich, Jablon, & Kark, 2015), and population viability analysis (Naujokaitis‐Lewis, Curtis, Arcese, & Rosenfeld, 2009). Testing the sensitivity of downstream management choices to upstream parameters will be an area for development in applied adaptive genomics.

## 4 EVALUATE AND ACT: ASSESSMENT

### 4.1 Evaluate the assessment

Next, the assessment should be interpreted in light of the conservation objectives and analytical limitations to determine whether the information is sufficient to inform conservation actions or whether further study is needed (Figure 1). Conclusions from the assessment may be equivocal, so a manager may decide to collect more data (i.e., sample more individuals, compare more populations, and sequence targeted genes; Figure 1). Alternatively, the assessment may clearly identify patterns of local adaptation and adaptive variants, providing the groundwork for initiating monitoring or conservation actions (e.g., identifying source populations for restoration, genetic rescue, or assisted gene flow). This will depend on the overall conservation plan and predefined thresholds for action.

In anonymous NGS studies, the number of candidate adaptive markers will be determined by the detection threshold, so this number is not reflective of the underlying processes but rather the chosen cutoff. While these methods are useful in detecting patterns of local adaptation, we caution against putting too much emphasis on any particular locus or set of loci identified (Pearse, 2016). Instead, broadscale patterns of geographic variation and relationships between genotypes and environmental drivers will be more informative, as will seeing if effects are localized on particular genomic regions (e.g., sex chromosomes). Another potential challenge for these studies is parallel evolution of adaptive traits via different genes and genetic architectures (Bernatchez, 2016; Ralph & Coop, 2015). This can confound sampling designs that are intended to improve the strength of inference by detecting local adaptation along replicated environmental gradients. In this case, the lack of a replicated signal of SNP–environment correlations does not necessarily mean that the detected signals are spurious, but may instead point to “imperfect” parallelism (Bernatchez, 2016). Finally, the differences in phenotypes underlying local adaptation are often the product of small changes in allele frequency across many genes, as well as the correlations among and interactions between these loci (Boyle, Yang, & Pritchard, 2017; Le Corre & Kremer, 2012). While different approaches may identify some of the same “core” genes involved (sensu Boyle et al., 2017), different subsets of the many “peripheral” genes will be detected with different sampling approaches and analytical methods. However, the patterns of variation identified will nonetheless provide important information for conservation actions.

Incorporating environmental data in GEA methods is a useful way to identify links between genetic mechanisms and environmental factors driving adaptation. However, it is important to remember that these studies cannot pinpoint causative relationships, as they are inherently correlative (Gunther & Coop, 2013). If it is necessary to identify a causative relationship before any management decisions can be made, then conducting experiments such as common gardens, genetic crosses, or genetic manipulations (e.g., gene editing or gene knockouts) will be required. Confirming causal relationships is very challenging, and to our knowledge has not been done for locally adaptive variants; nor is it necessary to inform conservation strategies for species in rapidly changing environments.

## 5 DESIGN AND IMPLEMENT: MONITORING

### 5.1 Design monitoring plan

Evaluating changes in genetic variation over time (e.g., detecting loss of genetic variability or changes in the frequencies of adaptive variants) requires a monitoring program. In an adaptive management context, monitoring is a means for both learning more about the system and evaluating the effectiveness of management actions once they are initiated (Lyons, Runge, Laskowski, & Kendall, 2008). While monitoring can include genetic or demographic assessments, in all cases effective monitoring programs identify threshold criteria for detecting biologically significant changes and spell out management interventions to be triggered by changes prior to initiating monitoring (Schwartz et al., 2007). Identifying **trigger points** can be challenging as threshold values are case‐dependent and likely differ among species (Atkinson et al., 2004). An effective approach is to set trigger points throughout the range of the **indicator variable** to ensure that management action is initiated before a crisis point is reached. Management interventions should be closely tied to the indicator variables, such that a triggered management action will directly affect the indicator and increase its value above the trigger point. For example, a continuous decline in allelic richness at putatively adaptive loci, or an observation of low survival or fecundity over multiple sampling periods may trigger a management intervention such as genetic rescue (Box 2) to increase allelic richness or fitness. By contrast, upgrading the species’ listing status would not directly impact the genetic indicator. Unfortunately, best practices for designing sampling protocols and interpreting genetic and other indicators for monitoring are sparse (more below). However, like other steps in the adaptive management framework, it is expected that monitoring plans will be adjusted to reflect new information (Section 6.1). This learning approach in the face of uncertainty best ensures that monitoring will trigger effective and timely management intervention, rather than simply documenting decline and “monitoring to extinction” (Lindenmayer, Piggott, & Wintle, 2013).

Monitoring panels of neutral and candidate adaptive markers can be developed from the initial genomic assessment using sequence capture or SNP arrays (Ali et al., 2015; Hoffberg et al., 2016; Jones & Good, 2016). These methods allow for consistent, efficient, and inexpensive genotyping of many individuals over time to inform diverse management objectives (Amish et al., 2012; Aykanat, Lindqvist, Pritchard, & Primmer, 2016; Hohenlohe, Amish, Catchen, Allendorf, & Luikart, 2011; Houston et al., 2014; Wright et al., 2015). This targeted approach to monitoring is preferred over repeated anonymous sequencing runs, as stochasticity inherent in that process will yield overlapping but distinct sets of loci. Targeted genotyping, by contrast, will optimize efforts by ensuring coverage of the same neutral and adaptive loci across multiple time points. Hess et al. (2015) provide a particularly good example of how a genomic assessment was effectively transitioned into a monitoring program for declining Pacific lamprey. Based on a genomic assessment (Hess, Campbell, Close, Docker, & Narum, 2013), they developed a SNP panel consisting of 96 neutral and candidate adaptive markers that were diagnostic for parentage analysis, cryptic species identification, and characterization of neutral and adaptive genetic variation. These SNPs were chosen to monitor the effectiveness of a diverse set of management actions including translocations, artificial propagation, and habitat restoration, as well as to track population size and facilitate species identification at early life stages. Adaptive markers linked to lamprey phenotypes (body size and migration timing) were included in the SNP panel to monitor the genetic basis of fitness‐related traits across different habitat types. Using one modest set of SNPs, the managers were therefore able to track fitness, population size, and individual movements to identify the success of conservation actions, which would have required much more intensive sampling and experimental work without the aid of genomics. However, because the number of adaptive markers (9) was very small in the monitoring panel, the authors warned against using these markers as an indication of overall adaptation, an important cautionary note when managing populations based on subsets of adaptive genetic variation.

Once the monitoring panel has been developed, the sampling design (number and distribution of samples) and temporal frequency of sampling must be designed to detect significant changes in allele frequencies or loss of adaptive variants in key populations (Allendorf, England, Luikart, Ritchie, & Ryman, 2008; Hoban et al., 2014; Schwartz et al., 2007). Because variation at neutral and adaptive loci is usually not correlated (Grueber, Hogg, Ivy, & Belov, 2015; Hartmann, Schaefer, & Segelbacher, 2014; Holderegger, Kamm, & Gugerli, 2006; Kremer et al., 2002), the appropriate number of loci and individuals monitored will depend on conservation objectives, biology of the organism, recent demographic history, and power of the genetic markers to detect change. While broad guidelines for demonstrating adaptive genetic changes have been outlined (Hansen, Sato, & Ruedy, 2012), little specific advice exists on temporal monitoring of adaptive variation (but see Landguth & Balkenhol, 2012). As a general rule, if the goal is to monitor change in allele frequency at a single locus, 30 individuals per population is often considered a sufficient sample size to detect an allele at a frequency of 5%; however, we suggest using simulations to determine a best sample size (Hale, Burg, & Steeves, 2012).

While simulations have been used for decades to aid in the development of genetic monitoring and the interpretation and evaluation of monitoring results (Palm, Laikre, Jorde, & Ryman, 2003; Waples, 2002; Waples & Teel, 1990), they have generally been underutilized for these purposes. Fortunately, user‐friendly simulation programs can be used to optimize sampling design and frequency to detect varying degrees of change. These can be customized to the biology of the focal species, seeded with current allele frequencies (Balkenhol & Landguth, 2011; Hoban, 2014), and parameterized for different outcomes in terms of selective changes or bottlenecks (Hoban, Gaggiotti, & Bertorelle, 2013a,b; Peery et al., 2012). Simulations can also be updated based on monitoring results to adjust trigger points and interventions and improve the effectiveness of management actions. Finally, simulations can be used to aid in the interpretation of genetic monitoring results. For example, Waples and Teel (1990) used simulations to test a set of potential drivers of substantial allele frequency changes in hatchery, but not wild, Pacific salmon populations. They were able to eliminate selection and admixture as potential causes and identify a low number of breeders per year as the driving factor.

### 5.2 Analyze monitoring data to detect temporal changes

In the case of both demographic monitoring and genomic monitoring, detecting temporal change depends on the frequency of sampling and the generation length of the organism. Monitoring data need to be analyzed regularly, on a timescale that is relevant to the indicator variable and the biology of the organism. For example, sampling allele frequencies multiple times within a single generation may confound changes in genetic structure across life history with changes across generations, whereas analyzing one age cohort in successive generations would be more informative. Monitoring data should be analyzed soon after collection to ensure the prompt detection of changes that might require conservation action. “Phase shifts,” sudden changes that occur with little warning (such as rapid declines in population status), are common aspects of biological changes, but some methods can help predict whether a phase shift is imminent (Dakos et al., 2012; Scheffer et al., 2009). Comparing change in adaptive markers to change in a reference set of selectively neutral markers can differentiate shifts due to genetic drift (which would affect all loci approximately equally) from those only occurring in candidate adaptive markers.

It may be necessary or useful in some cases to use museum or other historical ex situ samples (e.g., from a seed bank) to determine historical genetic variation conditions and compare those to contemporary and future changes (Bi et al., 2013; Hartmann et al., 2014; Larsson, Jansman, Segelbacher, Hoglund, & Koelewijn, 2008; Mikheyev, Tin, Arora, & Seeley, 2015; Schwartz et al., 2007). A disadvantage is that historical samples may not have all been collected at the same time or locations and may not have adequate sample sizes (which can reduce power) or DNA quality (which can cause errors). Regardless, keeping sample sizes consistent between sampled time points or adjusting estimates for sample size (e.g., through rarefaction) is important to maximize power to detect change (Dornelas et al., 2013). Sampling in excess of the target number of samples for monitoring is recommended (when feasible), as some samples may fail to be genotyped, and additional samples may be useful for some future objective (Schwartz et al., 2007).

## 6 EVALUATE AND ACT: MONITORING

### 6.1 Evaluate the monitoring results

Results from genetic monitoring should be evaluated in the context of the prespecified criteria for significant change: Have trigger points been met, and if so, when and how will management interventions be initiated? Do criteria indicate that a management intervention has been successful? If so, does the monitoring program need to be adjusted or discontinued? Do project objectives need to be revisited and updated? If the results are equivocal, what can be learned from the data to effectively adjust the monitoring plan (Figure 1)? For example, consider a management intervention of assisted gene flow has been implemented with the goal of introduced genotypes surviving and reproducing at least 5% more than local genotypes. If monitoring identifies that this threshold has been met, then the intervention is likely successful and should be continued or successfully concluded, whereas the reverse pattern would indicate that the assisted gene flow program needs adjustment or termination. While examples of genetic monitoring of this sort are currently scant, monitoring of phenotypes and reproductive rates has been used successfully in wolves and panthers (Hedrick & Fredrickson, 2010), and monitoring whether translocated individuals have reproduced is increasingly common (Koelewijn et al., 2010; Mulder et al., 2017). So far, temporal genetic monitoring of conservation interventions has been most widely used to understand the extent and efficacy of genetic rescue, including in bighorn sheep (Miller, Poissant, Hogg, & Coltman, 2012) and Florida panthers (Johnson et al., 2010).

When monitoring adaptive variation, unexpected outcomes may arise. One possibility is that a follow‐up study reveals some candidate loci are false positives or identifies additional adaptive markers. If this is the case, a revised set of adaptive markers will need to be included in genotyping and monitoring. Another possibility is that truly adaptive genetic variants are not changing in frequency, leading to the conclusion that the environment is not changing. However, genome complexity can constrain allele frequency changes in adaptive variants, even in changing environments, through antagonistic pleiotropy (one gene has multiple phenotypic effects, and positive effects of an allele on one trait are associated with negative effects on another), epistasis (a gene has a different phenotypic consequence when in a new genetic background due to interaction with another gene), or other evolutionary constraints (Hoffmann & Willi, 2008).

In all cases, data from genomic monitoring should be considered in the context of all available data for the species or population. For example, if demographic monitoring identifies population declines not reflected in the genetic data, the monitoring protocol and management strategies should be adjusted accordingly. Genetic indicators assess one aspect of a population (e.g., loss of genetic diversity) that is influenced by multiple ecological (population size, dispersal, breeding) and evolutionary processes (drift, migration, selection) that often interact. Therefore, interpreting causes of change (or lack thereof) in indicators over time may be challenging.

## 7 CONCLUSION

In this study, we present a modified adaptive management framework to help managers better understand the process of collecting NGS data and the potential applications for assessment and monitoring of adaptive variation (Figure 1). This framework emphasizes the iterative nature of adaptive management and highlights the importance of key decisions, particularly in the experimental design phase prior to the bulk of data collection (Figure 2). Considering the entire assessment and monitoring cycle prior to developing a project plan will enable researchers and managers to identify the scope of the project, clearly state assumptions and limitations of the chosen approach, and ensure that resources for the monitoring and action are available.

Assessing and monitoring adaptive and neutral genetic variation can be a powerful tool for conservation biologists and wildlife managers, but it has limitations. NGS is not a “silver bullet,” but it may be a useful tool, particularly when the entire adaptive management framework is considered prior to embarking upon a study, and with the understanding that implementation of management will be an iterative process that is likely to require adjustments and improvements over time.

## DATA ARCHIVING

There are no data associated with this review.
